# Histone deacetylase 6 in cancer

**DOI:** 10.1186/s13045-018-0654-9

**Published:** 2018-09-03

**Authors:** Ting Li, Chao Zhang, Shafat Hassan, Xinyue Liu, Fengju Song, Kexin Chen, Wei Zhang, Jilong Yang

**Affiliations:** 1 Department of Bone and Soft Tissue Tumor, Tianjin Medical University Cancer Institute and Hospital, Tianjin, 300060 People’s Republic of China; 2 National Clinical Research Center for Cancer, Key Laboratory of Cancer Prevention and Therapy, Tianjin’s Clinical Research Center for Cancer, Tianjin Medical University Cancer Institute and Hospital, Tianjin, 300060 People’s Republic of China; 3 International Medical School, Tianjin Medical University, Tianjin, 300061 People’s Republic of China; 4 Department of Epidemiology and Biostatistics, Tianjin Medical University Cancer Institute and Hospital, Tianjin, 300060 People’s Republic of China; 5 Cancer Genomics and Precision Medicine, Wake Forest Baptist Comprehensive Cancer Center, Winston-Salem, NC USA

**Keywords:** HDAC6, PD-1/PD-L1, α-tubulin, HSP90, Cortactin, Target therapy

## Abstract

Histone acetylation and deacetylation are important epigenetic mechanisms that regulate gene expression and transcription. Histone deacetylase 6 (HDAC6) is a unique member of the HDAC family that not only participates in histone acetylation and deacetylation but also targets several nonhistone substrates, such as α-tubulin, cortactin, and heat shock protein 90 (HSP90), to regulate cell proliferation, metastasis, invasion, and mitosis in tumors. Furthermore, HDAC6 also upregulates several critical factors in the immune system, such as program death receptor-1 (PD-1) and program death receptor ligand-1 (PD-L1) receptor, which are the main targets for cancer immunotherapy. Several selective HDAC6 inhibitors are currently in clinical trials for cancer treatment and bring hope for patients with malignant tumors. A fuller understanding of HDAC6 as a critical regulator of many cellular pathways will help further the development of targeted anti-HDAC6 therapies. Here, we review the unique features of HDAC6 and its role in cancer, which make HDAC6 an appealing drug target.

## Background

Tumorigenesis is a multistep process whereby normal cells are transformed into malignant cells, leading to an abnormal cell growth. Such transformational events are associated with major biological changes that are shared by most neoplastic cells, called hallmarks of cancer [1]. Mutations in key driver genes are the most potent contributors to cancer development. In addition to gene mutations, the deregulation of these epigenetic mechanisms (heritable changes in gene expression that do not involve DNA sequence modifications), including histone acetylation and deacetylation, is widely accepted to be an underlying cause of the cancer hallmarks [2]. As one of the key steps of post-translational modification in gene regulation and chromatin remodeling, the balance of acetylation and deacetylation of lysine residues is critical to maintaining body homeostasis, and disruption of this balance can contribute to the development of human diseases, including cancer [3–5].

As a key factor of histone acetylation and deacetylation, the expression of HDAC6 has been observed in normal heart, liver, kidney, testis, brain, and pancreas [6]. The increase of its expression or the destruction of its functional integrity can lead to a variety of diseases, such as Alzheimer [7, 8], Parkinson [9], and cardiovascular disease [10]. Meanwhile, the relationship between HDAC6 and tumors is also inseparable [11–13]. Some studies have reported the overexpression of HDAC6 in bladder cancer, malignant melanoma, and lung cancer [14–16]. While HDAC6 functions in deacetylating histones, recent reports have identified several critical nonhistone protein substrates for HDAC6, including α-tubulin, heat shock protein 90 (HSP90), and cortactin. HDAC6 can participate in the process of tumorigenesis and development through various pathways, such as oncogenic cell transformation and cancer cell migration and invasion [17–19]. In addition, the HDAC6 activity can affect the gene expression of some critical immune system molecules, including tumor-associated antigens, programmed death receptor-1 (PD-1), and programmed death receptor ligand-1 (PD-L1), which are central targets in cancer immunotherapy [20, 21].

Investigations of how HDAC6 regulates cancer-associated signaling pathways indicate that HDAC6 could be a potential therapeutic target for cancer patients [22–24]. Because of the unique molecular structure of HDAC6 and the diversity of its substrates, several groups have developed relatively isoform-specific inhibitors of its enzymatic action and some of these inhibitors are currently in clinical trials [25, 26]. In this review, we discuss how HDAC6 affects tumor development through its various substrates and the status of selected HDAC6 inhibitors.

## Structure of HDAC6 and differences from other HDACs

The histone deacetylase (HDAC) family in mammals contains 18 HDAC members that are grouped into four classes according to their homology to yeast deacetylases [27]. The class I HDACs, which include four isoforms (HDAC1, 2, 3, and 8); the class II HDACs, which contain HDAC4, 5, 6, 7, 9, and 10; and HDAC11, which is classified separately in class IV. All these three classes belong to Zn^+^-dependent proteases. However, the class III HDACs, which include Sirt1–7, work through NAD^+^-dependent mechanisms.

**Fig. 1:**
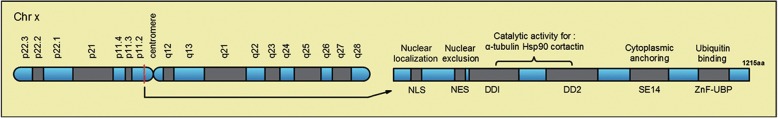
Structure of the HDAC6 gene and protein. The gene-encoding HDAC6 is located in Xp11.23 (left). The HDAC6 protein (right) contains two functional catalytic domains (DD1 and DD2), which catalyze deacetylation activity for α-tubulin, HSP90, and cortactin. The nuclear export signal (NES) promotes cytoplasmic localization of the protein, and the Ser-Glu-containing tetrapeptide (SE14) region ensures stable anchorage of the enzyme in the cytoplasm. Although the HDAC6 protein contains a nuclear localization sequence (NLS), HDAC6 mainly exists in the cytoplasm owing to the actions of the NES and SE14 motifs. The ubiquitin-binding zinc finger domain (ZnF-UBP domain, also known as the PAZ, BUZ, or DAUP domain) in its C-terminal region interacts with ubiquitinated proteins and mediates the regulation of ubiquitination-mediated degradation

HDAC6, a unique member of the type II HDACs, was first discovered by two different groups, Grozinger et al. [6] and Verdel and Khochbin [28]. The HDAC6 gene is located in Xp11.23 (Fig. 1) and encodes a protein of 1215 amino acids, the largest protein of the HDAC family. What is special about HDAC6 is that it contains two functional catalytic domains, and both domains are homologous and functionally independent of the overall activity of HDAC6 [6, 29–31]. Additionally, the C-terminal end of HDAC6 contains an ubiquitin-binding zinc finger domain (ZnF-UBP domain, also known as the PAZ, BUZ, or DAUP domain) that is related to the regulation of ubiquitination-mediated degradation [32]. Furthermore, unlike other HDACs that are localized in the nucleus [31], HDAC6 is mainly localized to the cytoplasm due to the presence of a nuclear export sequence (NES) and the SE14 motif, which is required for cytoplasmic retention (Fig. 1) [31, 33].

## Physiological function of HDAC6

Histone acetylation and deacetylation are among the key mechanisms of gene transcription regulation and are modified by histone acetyltransferases (HAT) and histone deacetylation enzyme (HDACs), respectively, leading to a complex chromosome configuration reconstruction and chromosome configuration change. HATs promote chromosome depolymerization and activate transcription. HDACs, on the other hand, block DNA and inhibit transcription. In addition to histones, HDAC6 also works on maintaining the acetylation balance of some nonhistone substrates, such as α-tubulin, cortactin, and HSP90 [14, 34, 35].

Microtubules (MTs) are key regulators of cell movement and are assembled by cytoplasmic α-tubulin. Notably, α-tubulin was the first identified nonhistone substrate of HDAC6, and the reversible deacetylation of α-tubulin by HDAC6 can affect MT stabilization and function [35, 36]. In addition, α-tubulin acetylation participates in mitotic events by affecting intracellular trafficking events through the protein encoded by the cylindromatosis gene (*CYLD*), which is essential for cell cycle progression [18, 37–39]. The N-terminal region of CYLD contains three CAP-Gly motifs, two of which (CAP-Gly1 and CAP-Gly2) associate directly with α-tubulin and promote tubulin polymerization [40].

**Fig. 2:**
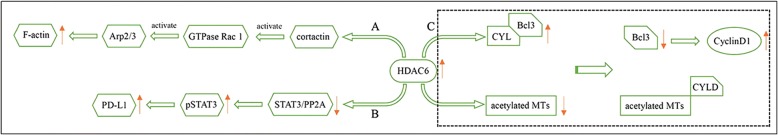
Targets and pathways of HDAC6. (A) HDAC6 binds to cortactin and induces its deacetylation, thereby activating the small GTPase Rac1 and actin-nucleated complex Arp2/3. Therefore, cortactin can be easily transferred to the cell edge and bind with F-actin to stimulate cell movement. (B) Under high levels of HDAC6, STAT3 is accumulated in its phosphorylation state, which reduces the interaction of STAT3 and PP2A. After entering into the nucleus, pSTAT3 and HDAC6 together bind the PD-L1 promoter to promote the expression of PD-L1. (C) In response to the high expression of HDAC6, the acetylation of MTs is decreased, and the interaction of CYLD and BCL3 is increased. CYLD is translocated to the cell periphery to bind acetylated microtubules, which allows BCL3 to enter into the nucleus, thereby promoting the expression of cyclin D1

In addition to its role in MT-dependent cell motility, HDAC6 also acts on another nonhistone substrate, cortactin, to influence actin-dependent cell motility [14, 15, 34, 39, 41]. As an F-actin-binding protein, cortactin promotes polymerization and branching, and it is usually found in areas of dynamic actin assembly, such as the leading edge of migrating cells [14, 37, 42]. Through binding to the deacetylase domains of HDAC6, cortactin is deacetylated [14]. Deacetylated cortactin then shows an increased ability to bind to F-actin through activating the small GTPase Rac1 and the actin-nucleating complex Arp2/3, thus promoting F-actin-dependent cell movement [14, 43] (Fig. 2). In contrast, when cortactin is highly acetylated, it cannot activate Rac1 or Arp2/3 and does not translocate to the cell periphery. With less cortactin in the cell periphery, the binding between cortactin and F-actin is decreased and cell motility is reduced [14, 43] (Fig. 2).

HSP90 is another nonhistone substrate of HDAC6, and its main function is to promote the maturation and maintenance of protein structures [44]. As the HSP90 deacetylation enzyme, HDAC6 can induce HSF1 activation, leading to the subsequent induction of molecular chaperone heat shock genes, including the gene-encoding HSP90. HDAC6 also directly interacts with HSP90 through its two catalytic domains and the ubiquitin zinc finger domain [34, 45], contributing to the deacetylation of HSP90 and interfering with its biological function, leading to a continued increase of its substrate proteins [46].

Importantly, the existence of the ZnF-UBP domain allows HDAC6 to function as a regulator of the ubiquitin and proteasome system (UPS), which regulates the cell response to protein misfolding [47]. After HDAC6 binding to ubiquitin-protein via its ZnF-UBP domain, the dynein protein-binding domain allows HDAC6 to bind to dynein and transport its misfolded proteins through microtubules to the perinuclear aggresomes. The polymers degrade misfolded proteins by autophagy [47]. On the one hand, HDAC6 promotes autophagy by recruiting and deacetylating cortactin, which is necessary for autophagosome and lysosomes. HDAC6, on the other hand, also forms complexes with HSP90 and HSF1, which are then involved in activating the heat shock transcription factor 1 (HSF1), inducing the expression of HSP25 and HSP70, guiding of protein folding, and participating in the repair and degradation of misfolded proteins [33].

## HDAC6 in cancer

### HDAC6 is required for oncogenic cell transformation

Anchorage-independent proliferation allows cells to survive by escaping anoikis, a special kind of programmed cell death that can result from the cell disengaging from the extracellular matrix and surrounding basement membrane [17]. A study performed by Lee et al. [17] indicated that HDAC6 promotes tumor formation and oncogenic transformation by facilitating anchorage-independent proliferation in transduced cells. First, the mouse embryonic fibroblasts (MEFs) derived from wild-type or HDAC6-null embryos were transduced with retrovirus-expressing SV40 early region and Ras^G12V^, which can transform cells from different sources into tumor cells, and then analyzed for anchorage-independent growth in soft agar. From the result, we can see that the number of colonies in the wild-type group was more than ten times that in the null group. Furthermore, knockdown of *HDAC6* in SKOV3 ovarian cancer, MCF7 breast cancer, and SKBR3 breast carcinoma cell lines reduced anchorage-independent growth to 3–20% [17]. To further validate these findings in vivo, stably expressed HDAC6-scrambled control and specific shRNA cells were injected independently into immunocompromised severe combined immunodeficient-Beige mice respectively. Two weeks later, the mice injected with HDAC6-shRNA showed fewer tumors than control mice [17].

Another interesting function of HDAC6 is found in the inflammatory breast cancer (IBC) cells. It is suggested that functional HDAC6 dependency is not only completely consistent with the change of protein expression but also related to the activity of HDAC6. Although HDAC6 is not overexpressed in the IBC cells, its activity is significantly higher in IBC cells compared with non-IBC cells [48]. The HDAC6 inhibitor ACY1215 (ricolinostat) can significantly inhibit the proliferation of IBC cells, both in vitro and in vivo, but it is less sensitive in non-IBC cells [48]. Therefore, HDAC6 functions in cancer cells not only involve alterations in its expression but also activities that control its cellular deacetylation. This represents a novel opportunity to develop therapeutic regimens specifically suited for IBC patients [48].

### HDAC6 modulates tumor development through nonhistone substrates

As described above, HDAC6 mainly participates in cell movement by acting on the nonhistone substrates. Increased cell mobility leads to MT depolymerization (i.e., de-adhesion events) as cells move and the remodeling of new adhesions at the continually forming front of the spreading cells. This process enhances tumor cell movement, metastasis, and invasion [27, 39]. In the research of Li et al., HDAC6 was highly expressed in human pancreatic cancer tissues at both the mRNA and protein levels, and the interaction of HDAC6 with cytoplasmic protein-170 increased cell motility, but had no obvious effect on pancreatic cancer cell proliferation and cell cycle progression [49]. As an estrogen-regulated gene, the expression of HDAC6 in estrogen receptor-positive breast cancer MCF-7 cells was also increased, and high HDAC6 expression increased cell motility by promoting HDAC6 binding to α-tubulin and enhancing MT activity [4, 50]. Consistently, cell motility studies in neuroblastoma showed that HDAC6 inhibitors decrease MT dynamics, leading to focal adhesion accumulation and reduced fibroblast motility [51].

In addition to cell motility, HDAC6 regulates the cell cycle through deacetylating α-tubulin and promoting the interaction of CYLD and BCL3 [18, 38] (Fig. 2). As reported, HDAC6 is highly expressed in malignant melanoma. When HDAC6 is silenced or knocked down, acetylated α-tubulin is increased, acetylated MTs are accumulated, and CYLD is translocated to the perinuclear region, leading to a reduced interaction between CYLD and BCL3 [18, 52, 53]. BCL3 is thus increased in the cytoplasm, and its transfer into the nucleus is decreased. Less BCL3 in the nucleus prevents the transcriptional activity of nuclear factor NF-ҡB, leading to the reduced expression of cyclin D1 and a significant delay of the cell cycle in the G_1_/S transition (Fig. 2) [18]. Thus, the regulation of α-tubulin by HDAC6 can enhance cell motility and mitosis, which in turn affects proliferation, metastasis, and invasion [18, 38, 54, 55].

The epidermal growth factor receptor (EGFR) and further activation of its downstream pathways lead to cell proliferation, especially in lung cancer [7, 8]. Therefore, affecting the synthesis and degradation of EGFR may affect the role of EGFR in tumors. Gao et al. reported that HDAC6 expression is closely involved in cell endocytosis and controls EGFR trafficking and degradation via deacetylation of α-tubulin [56]. With the loss of HDAC6, acetylated α-tubulin is accumulated, leading to the deregulation of microtubule-dependent endocytic vesicle trafficking and accelerating EGFR degradation [57–59].

Previous studies have demonstrated that HSP90 is essential for the stability and function of proteins that are involved in tumor metastasis [60], and HSP90 can affect the growth of tumor cells through stabilizing the levels of key chaperone proteins, especially AKT. HSP90 binding to AKT protects AKT from phosphates and thus maintaining AKT phosphorylation and activity. In turn, AKT binding of HSP90 protects HSP90 from degradation by proteasomes [34]. Further, as HSP90 affects the functional stability of AKT, it influences the PI3K/AKT signaling pathway, thereby affecting the cell survival, migration, differentiation, and angiogenesis [34, 61]. The targeted inhibition of HDAC6 also elevates acetylation of HSP90, which decreases the binding between HSP90 and ATP, thus reducing the combination of chaperone and oncogene [62], which could be of great importance in cancer treatment.

### Essential role of HDAC6 in the regulation of immunity in cancer

Immunotherapeutic strategies show great promise for cancer patients, especially those with tumors that lack molecular targets [63, 64]. Therefore, obtaining insights into the mechanism of immune tolerance may significantly improve the prognosis of patients.

HDAC6 has been shown to modulate the expression of specific tumor-associated antigens, MHC class I proteins, co-stimulatory molecules and cytokine production [20, 65]. In human melanoma cell lines, melanoma antigens *gp100*, *MART1*, *TYRP1*, and *TYRP2* were upregulated at the mRNA level following treatment with HDAC6 inhibitors (Nexturastat A or Tubastatin A). Protein expression of gp100 and MART1 also increased after genetic disruption of *HDAC6* [20]. Furthermore, HDAC6 seems to be an important regulator of the STAT3 pathway [66]. As an important transcriptional promoter, STAT3 is involved not only in the pathogenesis and sustainable development of many malignancies but also in the induction and maintenance of tumor immune tolerance [67]. Recent studies have clarified that STAT3 regulates the expression of PD-L1 in antigen-presenting cells as well as in a number of tumor cells, including lung cancer and melanoma, to inhibit the tumor immune response [21, 68]. Research by Woan et al. have also shown that HDAC6 can participate in antitumor immunity through STAT3-PD-L1 pathway [21]. High HDAC6 expression leads to STAT3 phosphorylation and ectopia into the nucleus at an invariant acetylation level and without an acetylation change in PP2A, Shp-2, and JAK2 proteins, which are directly involved in the homeostasis of phospho-STAT3 [69]. However, HDAC6 also reduces the interaction between STAT3 and PP2A, which is another mechanism affecting the activity of STAT3, thus phospho-STAT3 in the nucleus binding to its target genes and increasing their expression [21]. After entering the nucleus, pSTAT3 and HDAC6 are recruited to the promoter of PD-L1, where they activate PD-L1 gene transcription (Fig. 2). Conversely, upon knockdown of HDAC6, STAT3 was not detected at the PD-L1 promoter, indicating a requirement for HDAC6 in STAT3-mediated PD-L1 expression [21]. Furthermore, in antigen-presenting cells (APCs), HDAC6 binds STAT3 through the 503–840 amino acid region in HDAC6, and the complex then binds a specific sequence in the promoter of the anti-inflammatory and immunosuppressive cytokine IL-10 to increase its gene expression [70]. Decreased HDAC6 expression results in decreased IL-10 by reducing phosphorylation of STAT3 and the induction of inflammatory APCs that effectively activate antigen-specific naïve T cells and the reactive ability of CD4+ T cells [66, 70]. Together, these findings suggest that HDAC6 is indispensable in tumor immunity.

Meanwhile, recent preclinical trials have also demonstrated that the use of HDAC6 inhibitor ACY-241 in combination with a PD-L1 antibody enhances pDC-induced T- and NK cell-mediated cytolytic activities in multiple myeloma patient cells [71]. This provides a theoretical basis for the ongoing clinical trials combining a HDAC6 inhibitor with anti-PD-L1 treatment.

## HDAC6 inhibitors

### Anti-HDAC6 therapy in cancer pre-clinical trials

According to the different structure of HDAC inhibitors, they can be divided into four categories, namely, hydroxamates, cyclic peptides, aliphatic acids, and benzamides [72]. By regulating the acetylation state of histone, they interfere with the balance of histone acetylation and deacetylation in tumor cells; inhibit tumor cell angiogenesis [73], invasion, and metastasis [74, 75]; and induce apoptosis [76–78]. Due to the poor selectivity of HDAC inhibitor subtypes, they can lead to dose dependence and a series of toxic and side effects [79, 80]. Therefore, the research and development of a new selective HDAC inhibitor has an important role in improving the efficacy of anti-cancer treatment.

Sirtuins, as the only type of HDACs that plays a role through a NAD+-dependent mechanism, can maintain and renew mice hematopoietic stem cells, which provide a basis for selective inhibition and application [81]. For a new type of bendamustine-derived molecule with added HDACi activity, EDO-S101 is more effective than bendamustine and retained and increased the alkylation activity of deacetylase in multiple myeloma [82]. It can play a role in vitro in multiple myeloma cell lines and in vivo mice model by increasing the acetylation of α-tubulin and histones as well as the addition of potent DNA damage induction and impairment of DNA repair. Most importantly, EDO-S101 is the only single drug that has been shown to be effective in a multidrug-resistant Vk12653 mouse model [82]. All these findings provide the basis for clinical investigation of EDO-S101, either as a single agent or in combination with other chemotherapy drugs.

The first synthesized HDAC6-specific inhibitor was Tubacin [39]. However, the complexity of its synthesis and lipophilicity eventually prevented its use in vivo [39]. As the most commonly used HDAC6 inhibitor in clinical trials, ricolinostat has attracted wide clinical attention because it can inhibit the HDAC6-mediated aggregation pathway, increase the acetylation of tubule proteins, disrupt the transport of aggregates, and lead to protein aggregation and cell death accumulation [83]. At the same time, proteasome inhibits the misfolding/ubiquitination proteins produced by aggregation, which provide a rationale for the combination of proteasome inhibitors and HDAC6 inhibitors [84, 85]. In Jennifer E. et al.’s study on the effect and efficacy of the selective HDAC6 inhibitor ACY-1215 alone or combined with bortezomib in the preclinical model of lymphoma, ACY-1215 alone does cause increased acetylated α-tubulin, accumulated poly-ubiquitinated proteins, and upregulation of the unfolded protein response (UPR). In the apoptosis experiment, the highest apoptosis rate of OCI-LY10 cells occurred in 48 h after the treatment of IC50 (apoptosis induced by ACY-1215, bortezomib, or combined treatment were 9%, 20%, and 67%, respectively) [86]. The results demonstrated that the combination greatly improved the pharmacodynamic effects. This kind of combination has also led to a synergistic increase in poly-ubiquitinated proteins. Meanwhile, in a xenograft mouse model of diffuse large B cell lymphoma (DLBCL) in vivo, ACY-1215 plus bortezomib could significantly delay tumor growth and prolonged overall survival [86]. In another study, ACY-1215 could also accelerate the death of melanoma cells with a BRAF mutation caused by vemurafenib through inducing ER stress and inhibiting ERK activation [87], which provides a preclinical basis for the treatment of vemurafenib resistant malignant melanoma patients.

### Anti-HDAC6 therapy in cancer clinical trials

Structurally diverse HDAC inhibitors such as vorinostat (or suberoylanilide hydroxamic acid, SAHA) [88], romidepsin [89], belinostat [60], panobinostat [90], and chidamide [91] have recently been proposed as treatments for hematological malignancies and solid tumors. However, the non-selectivity of the HDAC inhibitors leads to obvious toxicity and side effects and limits the clinical application [92, 93].

According to a safety analysis from clinical trial of panobinostat in high-risk MDS or AML patients after allogeneic stem cell transplantation, 22/48 (52%) patients experienced at least one G3/4 adverse events (AEs) caused by panobinostat, the most common of which were thrombocytopenia (24%) and neutropenia (19%) (Table 1) [79]. Until the article is received, the median overall survival (OS) and relapse-free survival have not been reached after a median follow-up of 22 months [79]. This result was also consistent with another phase Ia/II panobinostat clinical trial, whose panobinostat-related G3/4 adverse events included thrombocytopenia (41.5%), fatigue (21%), and neutropenia (21%) [94]. In separate low-grade neuroendocrine tumors, although the adverse effects rate of panobinostat decreased, no patients showed a significant response with a 100% stable disease rate, and the median progression-free survival (PFS) was 9.9 months [95]. Similarly, for vorinostat, the high AE rate also limits its clinical dose [96, 97]. When combined with bortezomib, 16% G3/4 diarrhea, 22% G4 thrombocytopenia, and 17% G3/4 fatigue were observed [97].

**Table 1:** The comparison of results and adverse events among pan-HDAC inhibitors in clinical trials for cancer

NCT numbers	Agent	Other agents	Inclusion	Phase	Enrollment	mPFS (months)	AEs
NCT00985946	Panobinostat	–	Neuroendocrine tumors	II	15	9.9	Fatigue (27%)
							Thrombocytopenia (20%)
							Diarrhea (13%)
							Nausea (13%)
NCT01451268	Panobinostat	–	Myelodysplastic syndrome	I/II	62	–	Thrombocytopenia (24%)
			Acute myeloid leukemia				Neutropenia (19%)
NCT00918489	Vorinostat	–	Soft tissue sarcoma	II	40	3.2	Hematological toxicity (15%)
							Gastrointestinal disorders (13%)
							Fatigue (10%)
NCT01087554	Vorinostat	Sirolimus	Advanced cancer	I	249	2.1	Thrombocytopenia (31%)
		Everolimus					Neutropenia (8%)
		Temsirolimus					
NCT02944812	Chidamide	–	Peripheral T cell lymphoma	II	12	-	-
NCT02576496	EDO-S101	–	Hematological malignancies	I	84	-	-
			Multiple myeloma				
			Hodgkin’s lymphoma				
			Peripheral T cell lymphoma				
			Non-Hodgkin’s lymphoma				

*AEs* adverse effects

Therefore, more selective novel HDAC inhibitors may improve the prognosis of patients to a greater extent. For the novel subtype-selective HDAC inhibitors, chidamide selectively inhibits the activity of HDAC1, 2, 3, and 10 [91, 98]. The efficacy and safety have been demonstrated in a phase II clinical trial for relapsed or refractory peripheral T cell lymphoma (PTCL) [98]. In this study, the overall response rate (ORR) was 28% for all the T cell lymphoma patients. AITL patients, however, tended to have higher ORR (50%) rate. However, in the real-world, for the relapsed or refractory peripheral T cell lymphoma patients treated with chidamide, the ORR was 39.06%. When chidamide and chemotherapy are combined, ORR increases to 51.18% [91]. In addition, most AEs were of grade 1 to 2 [91].

As a special member of type II HDACs, the unique structure of HDAC6 also makes selective HDAC6 inhibitors a hot topic in clinical trials (Table 2). Ricolinostat (ACY-1215), a small selective HDAC6 inhibitor, is tolerated well as a monotherapy [99]. Ricolinostat with reduced class I HDAC activity, however, has minimal clinical activity as a single agent [99]. In a multicenter phase 1b clinical trial of ricolinostat together with lenalidomide and dexamethasone, ricolinostat (160 mg once daily on days 1–21 of a 28-day cycle as the recommended dose) combined with lenalidomide (25 mg) and dexamethasone (40 mg) were administered to 38 patients with relapsed or refractory multiple myeloma [100]. The response rate for all evaluable patients was 55% (21/38 patients [95% CI 38–71]), with low ricolinostat-related adverse events, according to the International Myeloma Working Group (IMWG) criteria. Notably, pharmacodynamics research results also showed that ricolinostat selectively inhibited HDAC6 with preserving the lower, tolerable level of class I HDAC inhibition, and the pharmacokinetics of ricolinostat and lenalidomide was not affected by co-administration at clinically relevant doses [100]. In another clinical trial of ricolinostat in the treatment of relapsed or refractory multiple myeloma, patients well tolerated a ricolinostat dose of 160 mg twice daily combined with bortezomib and dexamethasone treatment with a favorable toxicity rates (5% grade 3/4 diarrhea, 20% grade 3/4 thrombocytopenia, and 5% grade 3/4 fatigue) compared to those of vorinostat listed above [25]. Because the exposure doses of 160 mg and 240 mg per day showed no differences in pharmacological effects, ricolinostat at each of the two doses together with bortezomib and dexamethasone was studied. As a result, the overall response rate with the combination including daily ricolinostat at ≥ 160 mg was 37%, and the clinical benefit rate was 53% [25]. Together, these suggest that selective HDAC6 inhibitors combined with other chemotherapy drugs may show promise and benefit for cancer treatment.

**Table 2:** HDAC6 inhibitors in clinical trials for cancer

NCT numbers	Agent	Other agents	Inclusion	Phase	Start	End	Enrollment
NCT02935790	ACY-241	Nivolumab	Malignant melanoma	I	2016.10	2017.8	1
		Ipilimumab					
NCT02635061	ACY-241	Nivolumab	NSCLC	I	2015.12	2018.5	41
NCT01583283	ACY-1215	Lenalidomide	Multiple myeloma	I/II	2012.7	2018.11	38
		Dexamethasone					
NCT01323751	ACY-1215	–	Multiple myeloma	I/II	2011.3	2017.4	120
NCT02091063	ACY-1215	–	Lymphoma	I/II	2014.3	2017.5	40
			Lymphoid malignancies				
NCT02632071	ACY-1215	Nab-paclitaxel	Metastatic breast cancer	I	2015.12	2018.2	24
			Breast carcinoma				
NCT03008018	KA2507	–	Solid tumor	I	2017.8	2019.3	30

*NSCLS* non-small cell lung cancer

### Other epigenetic modification agents

In addition to histone modifications, epigenetic modifications also include DNA methylation and microRNAs [101]. As the most frequently studied epigenetic modification, methylation plays an important role in the molecular pathogenesis and prognosis of different types of cancers [102]. As for DNA methylation is closely related to the drug resistance of tumor cells, the use of epigenetic modification agents combined with chemotherapy can improve patients’ drug resistance [103]. For example, the prognosis of patients with malignant bone marrow tumors after allogeneic stem cell transplantation is still dismal. Considering the good efficacy and moderate toxicity of hypomethylated agents in the non-transplantation environment, the application of hypomethylated agents after transplantation has played a good role in preventing and treating recurrence [104].

Furthermore, the combination of microRNAs and other epigenetic drugs can also improve patient drug resistance [101]. Recent epigenetic studies have identified a group of tumor suppressor microRNAs, known as “epimiRNAs,” which were capable of regulating epigenetic modifications and being regulated by epigenetic modification, suggesting a regulatory circuit between microRNAs and epigenetic modification factors [105]. In multiple myeloma, mir-29b, as a common epimiRNA, can antagonize the carcinogenic effects of high HDAC4 expression [106]. In this interactive functional loop, silencing HDAC4 or using pan-HDAC inhibitor SAHA inhibits tumor cell growth and migration and increases cell apoptosis and autophagy. At the same time, the expression level of mir-29b was increased by promoter hyperacetylation. Similarly, the upregulation of mir-29b expression will increase the anti-tumor activity of SAHA, confirming the role of the HDAC4-mir-29b axis in regulating anti-myeloma drugs [106].

## Conclusion

The interaction of HDAC6 with histone and nonhistone substrates (such as α-tubulin, HSP90, and cortactin) is involved in gene transcription, DNA damage repair, and cell movement. Once the expression level of HDAC6 changes or its activity increases, it can lead to oncogenic cell transformation and tumor cell proliferation, invasion, metastasis, and mitosis. All these results provide the theoretical basis for the clinical application of HDAC6 inhibitors. The effects of HDAC6 on PD-L1 and the positive results of pre-clinical trials provide new ideas for the clinical application of HDAC6 inhibitors. Given the benign effects of HDAC6 inhibitors with lower adverse effects than HDAC inhibitors plus proteasome inhibitor treatment in hematological malignancies. Whether these therapies will be applied to other cancers remains to be seen. The present review expands our understanding of the current field and future directions and provides evidence that HDAC6, a cytosolic member of the HDAC family, may be an important target in anti-tumor strategies.

## Abbreviations

CYLD: Cylindromatosis gene
DLBCL: Diffuse large B cell lymphoma
F-actin: Filamentous actin
HAT: Histone acetyltransferase
HDAC6: Histone deacetylase 6
HSP90: Heat shock protein 90
IBC: Inflammatory breast cancer
MTs: Microtubules
PD-1: Program death receptor-1
PD-L1: Program death receptor ligand-1 receptor
PTCL: Peripheral T cell lymphoma
UPS: Ubiquitin and proteasome system
